# Zero-shot evaluation of ChatGPT for food named-entity recognition and linking

**DOI:** 10.3389/fnut.2024.1429259

**Published:** 2024-08-13

**Authors:** Matevž Ogrinc, Barbara Koroušić Seljak, Tome Eftimov

**Affiliations:** ^1^Jožef Stefan International Postgraduate School, Ljubljana, Slovenia; ^2^Department of Computer Systems, Jožef Stefan Institute, Ljubljana, Slovenia

**Keywords:** ChatGPT, food data, named-entity recognition, named-entity linking, natural language processing

## Abstract

**Introduction:**

Recognizing and extracting key information from textual data plays an important role in intelligent systems by maintaining up-to-date knowledge, reinforcing informed decision-making, question-answering, and more. It is especially apparent in the food domain, where critical information guides the decisions of nutritionists and clinicians. The information extraction process involves two natural language processing tasks named entity recognition—NER and named entity linking—NEL. With the emergence of large language models (LLMs), especially ChatGPT, many areas began incorporating its knowledge to reduce workloads or simplify tasks. In the field of food, however, we noticed an opportunity to involve ChatGPT in NER and NEL.

**Methods:**

To assess ChatGPT's capabilities, we have evaluated its two versions, ChatGPT-3.5 and ChatGPT-4, focusing on their performance across both NER and NEL tasks, emphasizing food-related data. To benchmark our results in the food domain, we also investigated its capabilities in a more broadly investigated biomedical domain. By evaluating its zero-shot capabilities, we were able to ascertain the strengths and weaknesses of the two versions of ChatGPT.

**Results:**

Despite being able to show promising results in NER compared to other models. When tasked with linking entities to their identifiers from semantic models ChatGPT's effectiveness falls drastically.

**Discussion:**

While the integration of ChatGPT holds potential across various fields, it is crucial to approach its use with caution, particularly in relying on its responses for critical decisions in food and bio-medicine.

## 1 Introduction

**Figure 1:**
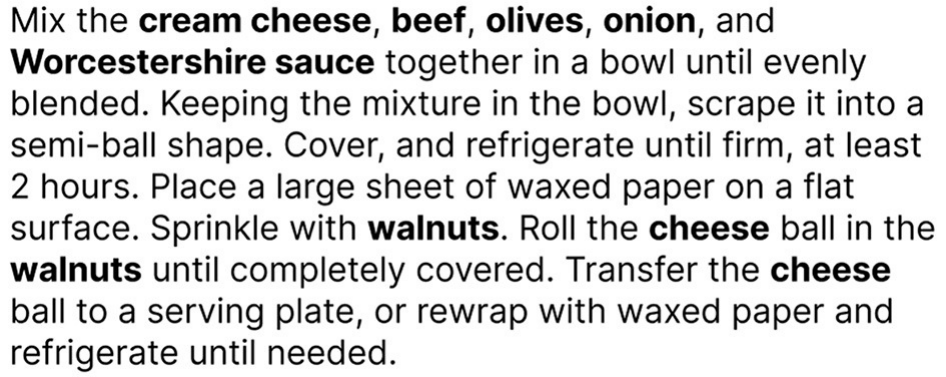
Food NER example from a recipe text.

Food has always been an important factor in our daily lives. Food can influence our health, mental health, fitness, and other aspects in conjunction with a person's well-being (1), but to understand the intricate relationship between food and healthcare, one needs to dig deep into the vast amount of scientific literature. As such, extracting food information from literature is crucial in ensuring that dietary choices are informed by rigorous research, promoting an accurate understanding of nutritional principles. Evidence-based dietary recommendations from scientific studies empower individuals to make informed choices, fostering a healthier lifestyle supported by robust scientific evidence (2). Yet, manual evaluation of such literature is a daunting task. Additionally, in digital dietary assessment, information on dietary habits is supplied in plain, unstructured text, and by automating food information extraction, we can assist clinicians and dietitians in improving a person's lifestyle and health. Structuring food information from unstructured text sources such as digital dietary assessments, recipes, and scientific literature involves two critical tasks in natural language processing (NLP): Named Entity Recognition (NER) (3) and named entity linking (4) (NEL). NER is a subtask of information extraction that automatically detects and categorizes entities (one or multiple words) from unstructured text. For instance, in Figure 1, an example of a recipe is presented, where the food entities are highlighted in bold and are automatically extracted by a NER method.

Depending on the methodology, several types of NER methods exist: dictionary-based, rule-based, corpus-based, active learning-based, and deep learning-based. Dictionary-based NERs are dependant on a pre-determined dictionary of the entities of interest (i.e., in our case, food entities) (5); rule-based NER also uses a pred-determined dictionary but in conjunction with rules that describe the characteristics of the entities in the domain of interest (6); corpus-based NERs are dependant on a corpus used to train a supervised machine learning model (7); active learning NERs use semi-supervised learning to train a model and further iteratively improve it using interactions from a user for new training instances (8), and deep learning-based NERs use large amounts of annotated data to train a model using deep neural networks (9). Despite many methodologies, the robustness and accuracy of a NER method is dependent on the amount of resources available for a specific domain.

**Figure 2:**
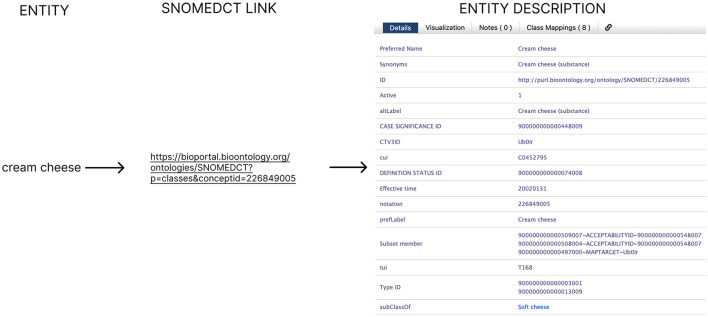
NEL example, where “cream cheese” is linked to the SNOMED-CT ontology.

NEL is the task of linking entities to their unique identifiers describing concepts in a knowledge base [i.e., in most cases, to a semantic model/ontology (10, 11)]. An ontology formally represents knowledge or concepts within a specific domain, detailing the entities, attributes, relationships, and constraints relevant to that domain. It serves as a structured framework for organizing and understanding information, facilitating knowledge sharing, reasoning, and interoperability between different systems and applications. Having a unique identifier helps us collect and comb information for the same entity from multiple sources (e.g., various scientific articles) even if it has a different textual representation (i.e. synonyms). This step is necessary to ensure that the data can interoperate effectively, which is crucial for adhering to the Findable, Accessible, Interoperable, and Reusable (FAIR) principles (12). In Figure 2, a NEL example is presented, where the first discovered entity “cream cheese” from the NER example (see Figure 1) is linked to the SNOMED-CT (13) ontology.

With the emerging development of generative artificial intelligence (AI) (14), particularly large language models (LLMs) [e.g., ChatGPT (15) LLaMA (16) Mistral (17), Gemini (18)], they offer a lot of potential in diverse NLP tasks, including NER and NEL.

**Our contribution:** This article delves into a zero-shot evaluation of the capabilities of ChatGPT-3.5 and ChatGPT-4 in the tasks of food NER and NEL, which are crucial for synthesizing data from diverse unstructured sources like academic literature and text in lay language. We explore ChatGPT's abilities, especially its capacity to perform these tasks without prior training (i.e., zero-shot evaluation), by curating a generalized prompt with which ChatGPT is capable of performing both tasks. Our primary goal is the evaluation of the task of food NER for which we utilized two food corpora. Next, we evaluate it on the task of food NEL by linking the recognized food entities to a unique identifier from the SNOMED-CT ontology or the FOODON ontology (19). Further, we perform a secondary evaluation of both models on the task of NER on biomedical domain entities and the task of NEL by linking them to the NCBI (20) ontology and MeSH ontology to compare how resource availability and entity types influence ChatGPT's performance on NER and NEL tasks.

## 2 Related work

In the domain of food NER and NEL, a lot of work has been done in the last decade to address the lack of annotated resources and NLP methodologies. Before the introduction of the FoodBase annotated corpora (21), most research focused on rule-based NER methods such as drNER (22) for knowledge extraction of evidence-based dietary recommendations and FoodIE (23) which is a rule-based NER method for food information extraction from recipes. Additionally, StandFood (24) has introduced a classification approach focusing on the lexical similarity of food entities that can be used for NEL of food entities to the FoodEx2 database provided by the European Food Safety Authority (EFSA). Following the introduction of FoodBase, further research using ML techniques has been introduced. In 2020 (25) released BuTTER, the first bidirectional LSTM for food NER utilizing the FoodBase annotated corpus to identify food entities. In the following year, FoodNER (26) was released as a fine-tuned bidirectional encoder representation from transformers model for food NER and NEL which can extract and annotate food entities in five different tasks and distinguish food entities on the level of food groups. To visualize and help food experts with the subject of different food standards and interoperability, FoodViz (27) has been developed, which is a web-based framework used to visualize and annotate food entities with semantic tags. Recent studies have also shown results on using deep learning architecture for food NER from recipes (28) and enhancing NER in agriculture by using LLMs (29).

In contrast to the food domain, the biomedical domain has experienced significant advancements over the past two decades, attributed to the large amounts of resources available. This development is predominantly focused on various biomedical entities, including genotypes, phenotypes, diseases, treatments, and drugs. To further advance NLP in this domain, multiple workshops have been organized, notably BioNLP (30), BioCreative (31), i2b2 (32), etc, each emphasizing biomedical data and excluding food entities. Additionally, multiple ontologies have been developed for the biomedical domain, SNOMED-CT, MeSH (33), Disease ontology (34), UMLS (35), which facilitate the organization and classification of biomedical entities. These advancements in the field have influenced the evolution of ML models with the most recent iteration of Bert (36), BioBert (37) and BioClinicalBERT (38), three examples of successful models in the field of biomedical NLP. With the introduction of LLMs, such as ChatGPT, researchers began testing its capabilities in the biomedical domain. Studies are focusing on improving LLMs for clinical NER via prompt engineering (39) and fine-tuning ChatGPT on biomedical NLP tasks instead of its zero-shot evaluation (40, 41). In addition, it has been highlighted that ChatGPT is effective in similar clinical NER tasks (42), even in zero-shot settings, despite trailing behind specialized models like BioClinicalBERT.

## 3 Materials and methods

Our study focuses on evaluating ChatGPT's capabilities in NER and NEL across two pivotal domains, food and biomedical. To achieve this, we utilize specially curated datasets. For the food domain, we used gold standard datasets from the European Food Safety Authority-funded project, encompassing a wide range of food-related data from scientific articles and food consumption data. While for the biomedical domain, we used chemical, disease and species corpora.

### 3.1 Food NER datasets

Our evaluation of the food domain utilizes two corpora from an EFSA-funded project, CAFETERIA. The first corpus is the CafeteriaSA corpus (43), comprised of 500 scientific abstracts, each annotated with food entities leading to a total of 6,407 annotations. These annotations include entities' unique identifiers from various semantic resources, including the Hansard taxonomy (44), FoodOn, and SNOMED-CT terminology. This corpus lays the foundation for extracting and comprehending food information from scientific texts. The second corpus is the CafeteriaFCD corpus (45), which extends the FoodBase corpus, annotating food consumption data (i.e. recipes) with unique identifiers from external resources such as Hansard taxonomy, FoodOn ontology, SNOMED-CT terminology, and the FoodEx2 (46) classification system. The CafeteriaFCD corpora is comprised of 1,000 recipes, each annotated with food entities, leading to a total of 7,429 annotations.

### 3.2 Biomedical NER and NEL datasets

Our evaluation in the biomedical domain incorporates three distinct corpora from two sources. The BioCreative V challenge (47) and the Linnaeus gold standard corpus (48). The BioCreative V Challenge is the fifth iteration of the Critical Assessment of Information Extraction Systems in Biology challenge. The event evaluates text mining and information extraction systems applied to the biological domain. One of the sub-tasks in the fifth edition of the challenge has been the evaluation of NER methodologies in the context of chemical entities and disease entities within life science literature with each entity linked (NEL) to its MeSH identifier. These two corpora have been further involved in our experiments. In addition, the Linnaeus corpus serves as a gold standard for species entity recognition, offering a comprehensive collection of annotations for species names within biomedical research texts. In contrast to the BioCreative V Challenge, the Linnaeus corpus uses the NCBI identifiers for the NEL task.

### 3.3 GPT models

In the exploration of ChatGPT's NER and NEL capabilities across the food and biomedical domains, our study employs two advanced iterations of the Generative Pre-trained Transformer models: ChatGPT-3.5 (49) and ChatGPT-4 (50). Each model iteration brings unique strengths to our experimental setup, allowing for an understanding of the evolution and applicability of these AI technologies in handling domain-specific entity recognition and linking tasks.

ChatGPT-3.5 represents an intermediate advancement in OpenAI's lineup of language models. Notably, it has been instrumental in setting benchmarks for language comprehension, context understanding, and the generation of human-like text based on the vast knowledge it has been trained on. Its application in our study serves as an evaluation of its capabilities, especially in processing and analyzing complex domain-specific text, providing critical insights into the limitations and strengths of AI-driven NER and NEL in the context of scientific and food consumption literature and as a benchmark for improvements in subsequent models.

ChatGPT-4, the subsequent iteration, builds upon the foundational successes of its predecessors, offering enhanced understanding and generation capabilities that promise significant advancements in AI's role within NER and NEL tasks. With a broader knowledge base and improved contextual awareness, ChatGPT-4 is designed to surpass the limitations observed in earlier models, providing more accurate entity recognition and linking across the specialized datasets utilized in our study. The introduction of ChatGPT-4 into our experimental workflow allows for a direct comparison of performance metrics with its predecessor in highlighting the progress made in language modeling and its practical implications for food and biomedical NER and NEL tasks.

## 4 Results

**Figure 3:**
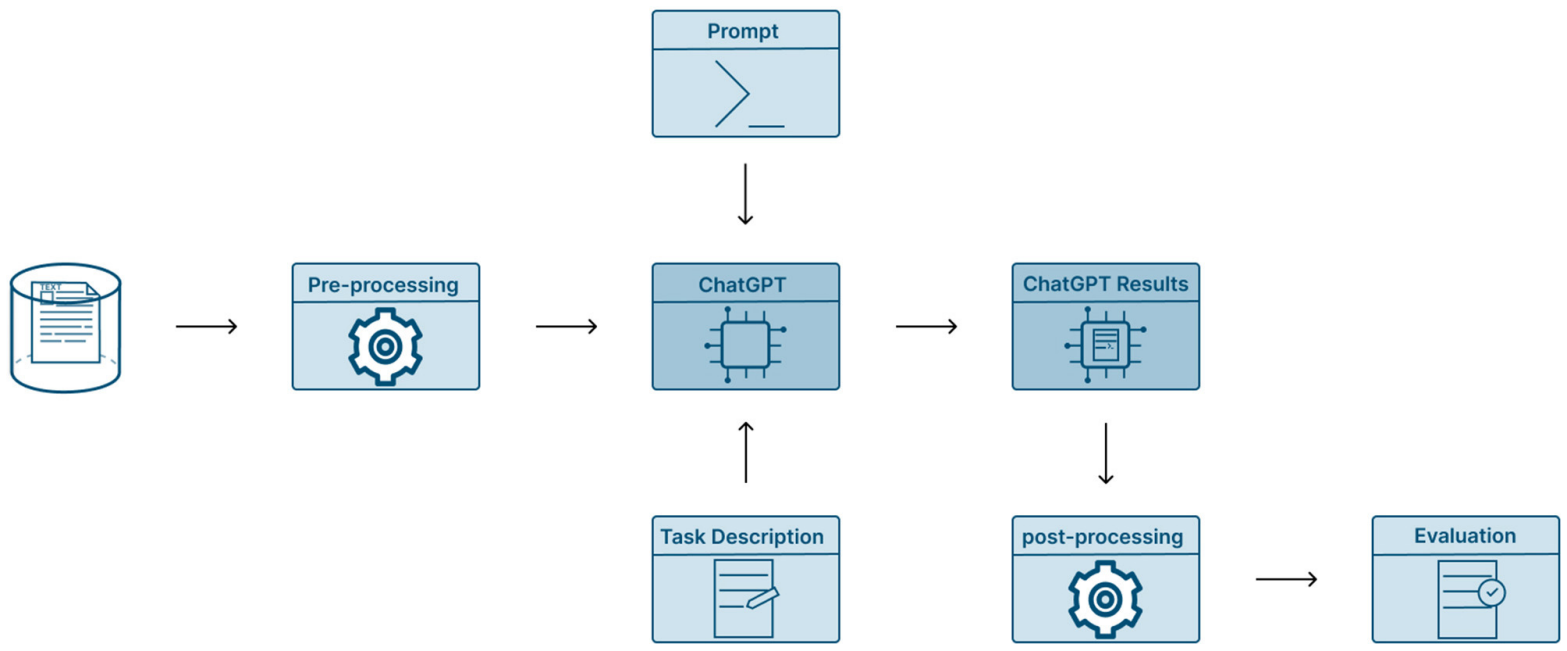
Pipeline for ChatGPT evaluation.

In Figure 3, our experimental flowchart is presented and modeled based on the approach in (51). The design of the prompt plays an important role in receiving suitable responses from ChatGPT. As such, we have created a general prompt to serve as a NER and NEL tool while incorporating essential elements such as contextual background, a clear task directive, and a specific output format constraint. In the following Table 1, we see an example of our prompt for each domain, which we used to extract and link entities.

**Table 1:** Prompts used to evaluate ChatGPT-3.5 and ChatGPT-4 on different domains.

**Domain**	**Prompt**
Food domain	*Extract only food entities and find their correct FOODON/SNOMED-CT ids, display the result only in this format name_of_FOOD|FOODON_id/SNOMEDCT_id without headers and nothing else. Text is:*
Chemical domain	*Extract only chemical entities and find their correct MeSH ids, display the result only in this format name_of_Chemical|MeSH_id without headers and nothing else. Text is:*
Disease domain	*Extract only disease entities and find their correct MeSH ids, display the result only in this format name_of_Disease|MeSH_id without headers and nothing else. Text is:*
Species domain	*Extract only species entities and find their correct NCBI ids, display the result only in this format name_of_Species|NCBI_id without headers and nothing else. Text is:*

The responses generated from the prompts proved to be sufficiently detailed for subsequent post-processing and analysis. To assess the performance we utilized the F1 score, a well-established metric combining precision and recall. The F1 metric is used to show the reliability of a model by calculating a score between 0 and 1. A score higher than 0.9 indicates excellent performance. A score between 0.8 and 0.9 is considered good, while a score between 0.5 and 0.8 is average. An F1 score below 0.5 is considered poor performance. The F1 score calculation (Equation 1) is calculated using precision (Equation 2) and recall (Equation 3). TP means true positive or correctly found entities, FP means false positive or entities that have been found but are incorrect, and FN means false negative, entities that have not been found.


(1) $$\begin{array}{l} F1\;score=\frac{2*Precision*Recall}{Precision+Recall} \end{array}$$

(2) $$\begin{array}{l} Precision=\frac{TP}{TP+FP} \end{array}$$

(3) $$\begin{array}{l} Recall=\frac{TP}{TP+FN} \end{array}$$

### 4.1 Food NER and NEL evaluation

To enhance accuracy and minimize ChatGPT's generation of erroneous information in the food domain, we define our initial prompt to align with the specific requirements of the Cafeteria corpora. An example of the text used with the prompt is seen with the response received from ChatGPT-4 in Table 2.

**Table 2:** ChatGPT-4 response for food entities.

Example text	“Mix the cream cheese, beef, olives, onion, and Worcestershire sauce together in a bowl until evenly blended.”
Domain	ChatGPT-4 response
Food	“cream cheese|762563006 beef|767623000 olives|722867003 onion|769846004 Worcestershire sauce|771471005”

After analyzing the responses, we noticed instances where ChatGPT identified food entities as partial matches, omitting prefixes or suffixes. For example, instead of recognizing PROVOLONE CHEESE in its entirety, ChatGPT identified only CHEESE. Initially categorized as false positives, these partial matches made us reconsider our evaluation criteria. Consequently, we explored whether acknowledging partial matches as correct could enhance the model's performance assessment, shifting from viewing them strictly as errors to potential positives.

### 4.2 NER

**Figure 4:**
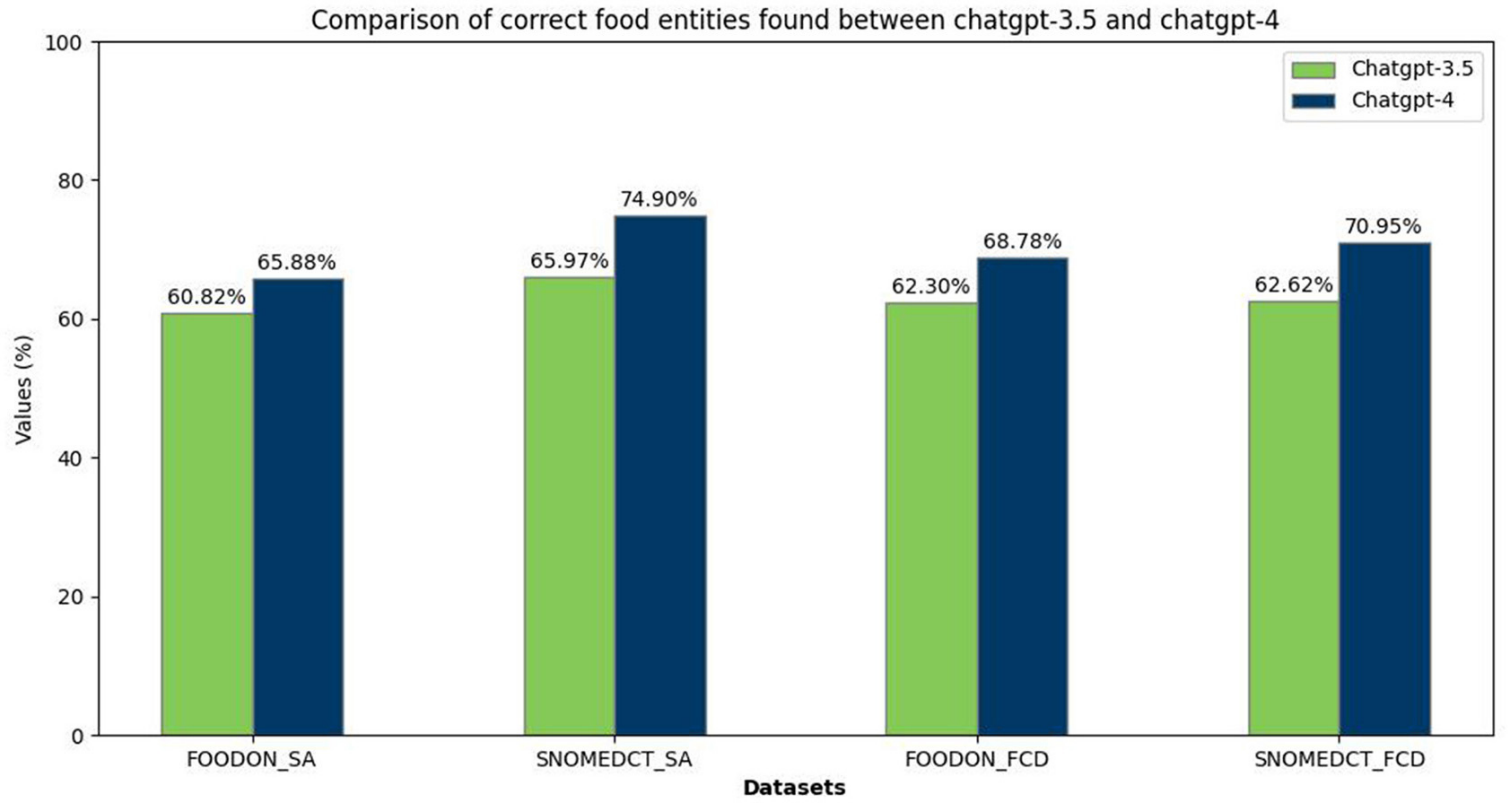
Comparison between ChatGPT-3.5 and ChatGPT-4 in finding correct food entities.

In the NER task, ChatGPT demonstrated good performance across both culinary recipes (CafeteriaFCD) and scientific articles (CafeteriaSA). Figure 4 illustrates the quantity of precisely identified food entities, with an entity deemed accurate only if identified with 100% correctness (not a partial match). The labels FOODON/SNOMED-CT indicate text documents annotated using FOODON or SNOMED-CT identifiers, whereas SA/FCD represents scientific articles or food consumption data. Observations reveal that ChatGPT-3.5 and ChatGPT-4 exhibit greater proficiency in identifying food entities within food consumption data (CafeteriaFCD) than scientific articles (CafeteriaSA).

Moreover, there is a marginally higher performance level in ChatGPT-4 relative to ChatGPT-3.5, which aligns with expectations considering its status as an enhanced model. However, the performance gap between the two versions is surprisingly narrow. Upon reviewing the F1 scores presented in Table 3, it becomes evident that ChatGPT-4 slightly outperforms ChatGPT-3.5 in the accuracy of food entity identification, particularly in culinary recipes over scientific texts. Furthermore, the disparity in performance between ChatGPT-3.5 and ChatGPT-4 is more evident in the context of scientific articles than in food recipes, underscoring the improvements in the latest model's capabilities. In comparison, if we add partial matches to our evaluation, the performance of both models rises, which illustrates that although ChatGPT demonstrates proficiency in food domain NER tasks, its inability to detect prefixes and suffixes, critical elements that significantly impact food identification, may lead to confusion when determining the precise type or brand of food. If we compare our findings with FoodNER and SciFoodNER (52), taking into consideration that both models are fine-tuned to different domain-specific data, FoodNER on food consumption data and SciFoodNER on scientific articles, we notice that both ChatGPT-3.5 and ChatGPT-4 fall behind. In comparison to FoodNER, the F1 score of ChatGPT-4 partials on FOODON FCD came the closest with a difference of 0.104, while in contrast to SciFoodNER, ChatGPT-4 partials on FOODON SA came the closest with a more significant difference of 0.201.

**Table 3:** F1 scores for ChatGPT-3.5 and ChatGPT-4 for food domain (NER task).

**Model**	**FOODON SA**	**SNOMED-CT SA**	**FOODON FCD**	**SNOMED-CT FCD**
ChatGPT-3.5	0.404	0.363	0.619	0.520
ChatGPT-3.5 partials	0.539	0.463	0.786	0.708
ChatGPT-4	0.533	0.490	0.668	0.562
ChatGPT-4 partials	0.699	0.604	0.839	0.749

### 4.3 NEL

For the NEL task, we assessed the performance of both models in accurately associating food entities with their respective SNOMED-CT or FOODON identifiers. Each identifier is a unique code corresponding to a specific food entity. For example, CHEESE is represented with a FOODON identifier of 00001013 and with a SNOMED-CT identifier of 102264005. According to the results, the outcome fell short of our expectations. Apart from ChatGPT-4 successfully linking only two correct identifiers within the FOODON CafeteriaFCD corpus, neither model could associate any food entity with its corresponding identifier. Given these results, calculating the F1 score for this task was deemed unnecessary. Alternatively to ChatGPT's performance, FoodNER models achieved a macro F1 score between 0.733 and 0.789 on food consumption data, while SciFoodNER models achieved a median macro F1 score of around 0.42. Illustrating the difficulty of NEL in the domain of food.

### 4.4 Biomedical NER and NEL evaluation

To compare our findings within the food domain, we have extended our analysis to a more established area of study, the biomedical domain. Our examination in this domain drew upon three previously mentioned corpora, two from the BioCreative V challenge and one from the Linnaeus corpus. We adapted our prompt for each corpus by modifying the original to suit the specific corpus focus. For the chemical and disease entities corpora, which use MeSH identifiers, the prompt has been tailored to extract the relevant entities and their corresponding MeSH IDs. For instance in Table 4, we see responses from ChatGPT-4 for the example text.

**Table 4:** ChatGPT-4 response for chemical and disease entities.

Example text	“Famotidine is a histamine H2-receptor antagonist used in inpatient settings for prevention of stress ulcers and is showing increasing popularity because of its low cost”
Domain	ChatGPT-4 response
Disease	“stress ulcers|D004487”
Chemical	“Famotidine|D005242 Histamine H2-Receptor Antagonists|D006632 Stress Ulcers|D013379”

For the Linnaeus dataset, which catalogs species entities using NCBI identifiers, we used a prompt designed for extracting species entities and their accurate NCBI IDs. An example of the response and text is seen in Table 5.

**Table 5:** ChatGPT-4 response for species entities.

Example text	“including the ribosomal protein S3 from Escherichia coli (1), Mer1p from S.cerevisiae, a meiosis-specific splicing factor (1), MEX-3 from Caenorhabditis elegans, presumably involved in mRNA localization during development (2)”
Domain	ChatGPT-4 response
Species	“Escherichia coli|562 Caenorhabditis elegans|6239”

### 4.5 NER

**Figure 5:**
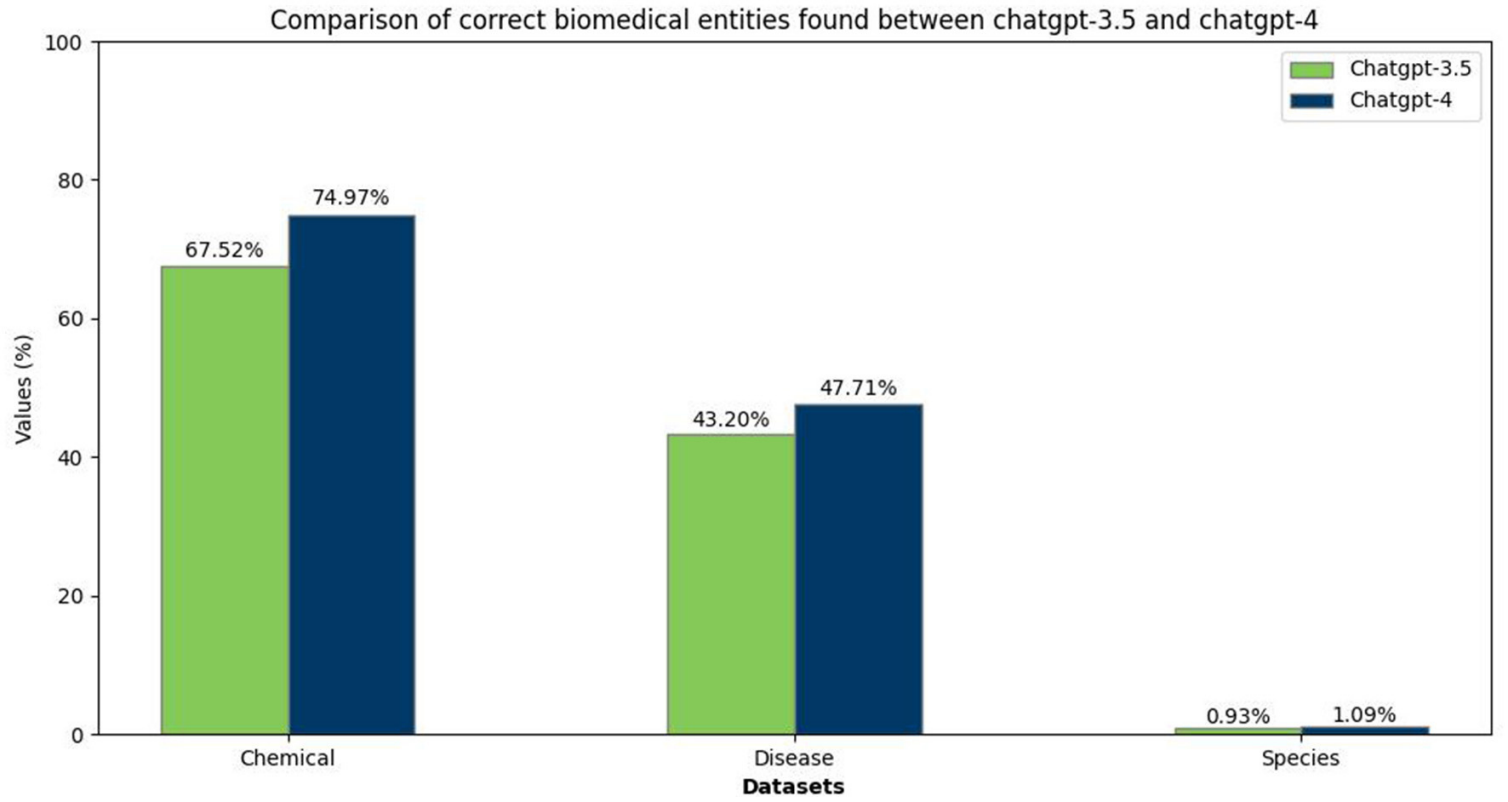
Comparison between ChatGPT-3.5 and ChatGPT-4 in finding biomedical entities.

For the NER task, the performance outcomes of both ChatGPT-3.5 and ChatGPT-4 are depicted in Figure 5. This figure indicates that the models perform better in identifying chemical and disease entities while struggling with species entities. This disparity could be attributed to the narrower scope of chemical and disease terminology instead of the broad and varied taxonomy of species. The observation shows the challenge in species entity recognition, reflected in both models' performance metrics.

The F1 scores in Table 6 display the same pattern. The task of identifying chemical and disease entities proved to be easier, while recognizing species posed a more significant challenge. Adding to this, our findings from the food domain, the incorporation of partial matches significantly enhanced the performance of both models across the chemical and disease datasets and, to a lesser extent, for the species dataset. In addition, the results correspond to those reported in articles (51, 53) with a little margin of difference.

**Table 6:** F1 scores for ChatGPT-3.5 and ChatGPT-4 for the biomedical domain (NER task).

**Model**	**Chemical**	**Disease**	**Species**
ChatGPT-3.5	0.578	0.404	0.016
ChatGPT-3.5 partials	0.646	0.535	0.021
ChatGPT-4	0.698	0.514	0.021
ChatGPT-4 partials	0.772	0.692	0.023

### 4.6 NEL

**Figure 6:**
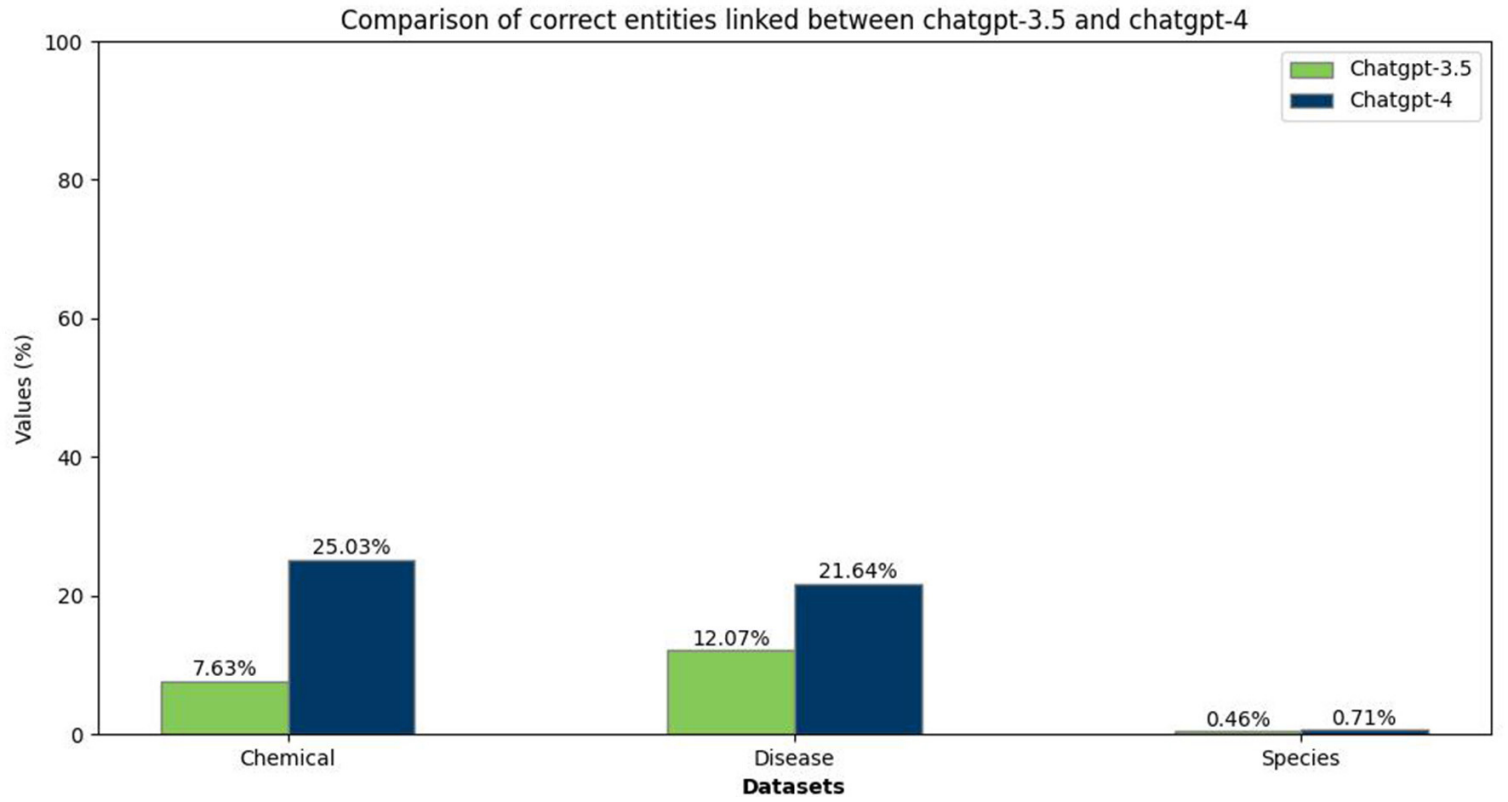
Comparison between ChatGPT-3.5 and ChatGPT-4 in linking biomedical entities to identifiers.

In our analysis of the NEL task within the biomedical domain, we have evaluated the performance of both ChatGPT-3.5 and ChatGPT-4 in mapping chemical, disease, and species entities to their respective MeSH or NCBI identifiers, paralleling our approach in the food domain. The outcomes, as depicted in Figure 6, revealed a marked superiority of ChatGPT-4 over ChatGPT-3.5. Furthermore, accurately linking disease and chemical entities proved challenging for both models, demonstrating the complexity of named entity linking despite the well-documented and recognized datasets.

The F1 scores in the accompanying Table 7 confirm ChatGPT-4 enhanced proficiency in linking entities across all categories compared to ChatGPT-3.5. Additionally, ChatGPT-4 exhibits a notable advancement in identifying chemical and disease entities. However, the overall performance, particularly in species entity linking, remains sub-optimal, highlighting areas for future model refinement and research focus.

**Table 7:** F1 scores for ChatGPT-3.5 and ChatGPT-4 for biomedical domain (NEL task).

**Model**	**Chemical**	**Disease**	**Species**
ChatGPT-3.5	0.065	0.113	0.008
ChatGPT-4	0.233	0.233	0.0139

## 5 Discussion

We observe that in food NER, ChatGPT models perform comparably to FoodNER and BuTTER, which are fine-tuned on specific datasets. This suggests that GPT models can be applied to food NER tasks but incur financial costs. Conversely, developing specialized food NER models like FoodNER and BuTTER involves extensive manual data annotation, leading to a time-consuming process. In addition, ChatGPT models are not effective for food NEL without fine-tuning.

The tasks of NER and NEL are essential for making data interoperable, which is especially apparent in the food domain and is a crucial part of accomplishing FAIR principles. Additionally, these tasks open up the ability to link food data to biomedical data, where data normalization is necessary and can be achieved by performing the tasks presented in this study. By facilitating the linkage of food data to biomedical data, these tasks empower professionals to make well-informed decisions regarding patient care, dietary recommendations, and overall health management. The normalization of data achieved through NER and NEL offers valuable insights that can directly impact clinical and nutritional assessments, fostering more precise and personalized interventions. While ChatGPT-4 yields promising results in the task of NER, its shortcomings in the task of NEL leave much room for improvement before becoming a reliable method for accomplishing both tasks.

## 6 Conclusion

In our study, we evaluated the performance of ChatGPT versions 3.5 and 4 on NER and NEL tasks within food and biomedical domains. After testing multiple prompt designs, we found a general prompt that was the most effective and least costly in retrieving results from both ChatGPT-3.5 and ChatGPT-4. ChatGPT-4 showed a slight edge over ChatGPT-3.5, especially in identifying entities in food consumption data (FCD) versus scientific articles (SA). Incorporating partial matches significantly improved both models' performance, suggesting a refined approach to entity recognition. Unfortunately, the performance of ChatGPT on NEL in the food domain highlights ChatGPT's lack of information for this particular task. In the biomedical domain, similar performance trends were observed, with ChatGPT-4 outperforming ChatGPT-3.5 in mapping entities to MeSH or NCBI identifiers. Despite yielding better results in linking disease and chemical entities, NEL proves a difficult challenge for both models. Additionally, ChatGPT's performance in species entity recognition from the Linnaeus dataset aligns closer to the results from the food domain. The comparison to specialized models discussed in related literature for the biomedical domain (54) and for the food domain (25) indicates specialized models' superiority in specific NER tasks. Yet, compared to models not trained with the same dataset or out-of-corpus (OOC), ChatGPT's performance aligns more closely, showcasing its potential adaptability (i.e., fine-tuning), which should be considered for future work.

## Data availability statement

The original contributions presented in the study are included in the article/supplementary material, further inquiries can be directed to the corresponding author.

## Author contributions

MO: Data curation, Formal analysis, Methodology, Software, Validation, Visualization, Writing – original draft. BK: Conceptualization, Writing – review & editing. TE: Conceptualization, Methodology, Project administration, Supervision, Writing – review & editing.
